# Metabolomics for Age Discrimination of Ginseng Using a Multiplex Approach to HR-MAS NMR Spectroscopy, UPLC–QTOF/MS, and GC × GC–TOF/MS

**DOI:** 10.3390/molecules24132381

**Published:** 2019-06-27

**Authors:** Dahye Yoon, Bo-Ram Choi, Seohee Ma, Jae Won Lee, Ick-Hyun Jo, Young-Seob Lee, Geum-Soog Kim, Suhkmann Kim, Dae Young Lee

**Affiliations:** 1 Department of Herbal Crop Research, National Institute of Horticultural and Herbal Science, RDA, Eumseong 27709, Korea; 2 Department of Chemistry, Center for Proteome Biophysics and Chemistry Institute for Functional Materials, Pusan National University, Busan 46241, Korea

**Keywords:** ginseng, age discrimination, HR-MAS NMR, UPLC–QTOF/MS, GC × GC–TOF/MS

## Abstract

(1) Background: The ability to determine the age of ginseng is very important because the price of ginseng depends on the cultivation period. Since morphological observation is subjective, a new scientific and systematic method for determining the age of ginseng is required. (2) Methods: Three techniques were used for a metabolomics approach. High-resolution magic-angle-spinning nuclear magnetic resonance (HR-MAS NMR) spectroscopy was used to analyze powdered ginseng samples without extraction. Ultrahigh-performance liquid chromatography quadrupole time-of-flight mass spectrometry (UPLC-QTOF/MS) and gas chromatography quadrupole time-of-fight mass spectrometry (GC-TOF/MS) were used to analyze the extracts of 4-, 5-, and 6-year-old ginseng. (3) Results: A metabolomics approach has the potential to discriminate the age of ginseng. Among the primary metabolites detected from NMR spectroscopy, the levels of fumarate and choline showed moderate prediction with an area under the curve (AUC) value of more than 0.7. As a result of UPLC-QTOF/MS-based profiling, 61 metabolites referring to the VIP (variable importance in the projection) score contributed to discriminating the age of ginseng. The results of GC×GC-TOF/MS showed clear discrimination of 4-, 5-, and 6-year-old ginseng using orthogonal partial least-squares discriminant analysis (OPLS-DA) to 100% of the discrimination rate. The results of receiver operating characteristic (ROC) analysis, 16 metabolites between 4- and 5-year-old ginseng, and 18 metabolites between 5- and 6-year-old ginseng contributed to age discrimination in all regions. (4) Conclusions: These results showed that metabolic profiling and multivariate statistical analyses can distinguish the age of ginseng. Especially, it is meaningful that ginseng samples from different areas had the same metabolites for age discrimination. In future studies, it will be necessary to identify the unknown variables and to collaboratively study with other fields the biochemistry of aging in ginseng.

## 1. Introduction

Ginseng has been used as a natural medicinal ingredient in East Asia for thousands of years. It is a perennial crop, and 4–6-year-old ginseng is used in many medicines [1]. Ginseng’s medicinal properties are dependent on the cultivation period, and 6-year-old ginseng is considered to be the most effective [2]. Although 6-year-old ginseng only accounts for about 10% of the total ginseng production in Korea, it is readily available in markets. However, this may be because some 4- and 5-year-old ginseng is sold as 6-year-old ginseng. Therefore, as the price of ginseng is dependent on the cultivation period, the ability to determine its age is necessary to correct the turbulent market. Previous studies have analyzed the saponin content in ginseng extracts [3] and recorded observations of ginseng’s morphology, including the roots, stems, stem scars, rhizomes, secretory ducts, and growth rings [4,5]. Since morphological observation is subjective and the extraction process lowers test reproducibility, new scientific and systematic methods of determining the age of ginseng are required.

The objective of this study was to distinguish the age of ginseng based on metabolomics. We selected metabolomics as a novel approach to identifying changes in the metabolic profiles. Metabolites are the end products in vivo that are close to the phenotype [6]. Our hypothesis was that the metabolic profiles may vary in predictable ways according to cultivation region, ginseng’s age, and other external factors. Previous studies on discriminating the age of ginseng by metabolomics used samples from a single area [3,7,8,9,10,11,12]. However, because there is a difference between regions, age discrimination should be done for samples obtained from various areas.

Metabolomics studies allow the use of a whole metabolome in a sample by comparing and analyzing changes in the metabolite profile rather than by comparing the concentrations of each metabolite. Therefore, studies based on metabolomics do not aim to discover specific biomarkers but rather to identify the metabolite profile [13].

Primary metabolites, such as proteins, carbohydrates, nucleic acids, and lipids, are involved in the performance of physiological functions in the cell [14]. Plants produce not only primary metabolites but also secondary metabolites that protect them [15]. Most ginseng studies have been conducted on these secondary metabolites regarding the roles of biomarkers and medicinal properties [16,17,18].

In this study, both the primary and secondary metabolites of a ginseng were targeted for profiling by using high-resolution magic-angle-spinning nuclear magnetic resonance (HR-MAS NMR) spectroscopy, ultrahigh-performance liquid chromatography quadrupole time-of-flight mass spectrometry (UPLC-QTOF/MS), and gas chromatography quadrupole time-of-fight mass spectrometry (GC-TOF/MS), respectively. For the simultaneous detection of all metabolites in ginseng, a multiplex approach was necessary. Metabolomics studies were carried out to distinguish the age of ginseng by combining the advantages of the three techniques. The profiling results were used to discriminate the age of ginseng through multivariate statistical analyses, including principal component analysis (PCA), partial least-squares discriminant analysis (PLS-DA), and orthogonal partial least-squares discriminant analysis (OPLS-DA), which were applied to find chemical markers to determine the age of ginseng.

## 2. Results

### 2.1. HR-MAS NMR-Based Metabolomics Analysis to Discriminate the Age of Ginseng

**Figure 1:**
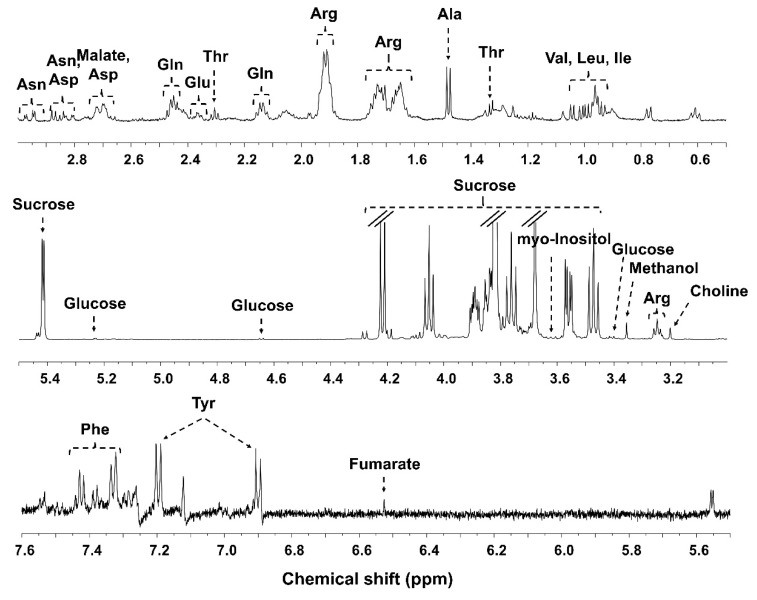
Representative High-resolution nuclear magnetic resonance (^1^H-NMR) spectrum and annotation of major metabolites of powdered ginseng acquired from 600 MHz nuclear magnetic resonance (NMR) spectroscopy.

All of the detected primary metabolites were assigned and quantified in each NMR spectrum. A representative ^1^H-NMR spectrum is shown in Figure 1 and the main metabolites are annotated. The main primary metabolite was sucrose, and other primary metabolites such as glucose, organic acids, and amino acids were found in the ginseng. Representative ^1^H-NMR spectra of each age are shown in Figure S1. Metabolites in the NMR spectra were quantified referring to 3-(trimethylsilyl) propionic-2,2,3,3-*d*_4_ acid sodium salt (TSP-*d*_4_) (2 mM) using Chenomx Suite software. The fold changes of all metabolite concentrations were calculated and univariate statistical analyses were conducted to confirm the significance (Table 1). There were more metabolites showing the opposite tendency between the change pattern of 4–5-year-old ginseng and 5–6-year-old ginseng. Proline was decreased in both 5- and 6-year-old ginseng. Phosphocholine, threonine, tyrosine, and asparagine were increased in both 5- and 6-year-old ginseng. Fumarate, valine, 4-aminobutyrate, sucrose, isoleucine, leucine, glutamine, phenylalanine, choline, arginine, alanine, and ethanolamine were decreased in 5-year-old ginseng, but they were increased in 6-year-old ginseng. On the other hand, malate, glycerophosphocholine, inositol, glutamate, glucose, and aspartate were increased in 5-year-old ginseng, while they were decreased in 6-year-old ginseng. 

**Figure 2:**
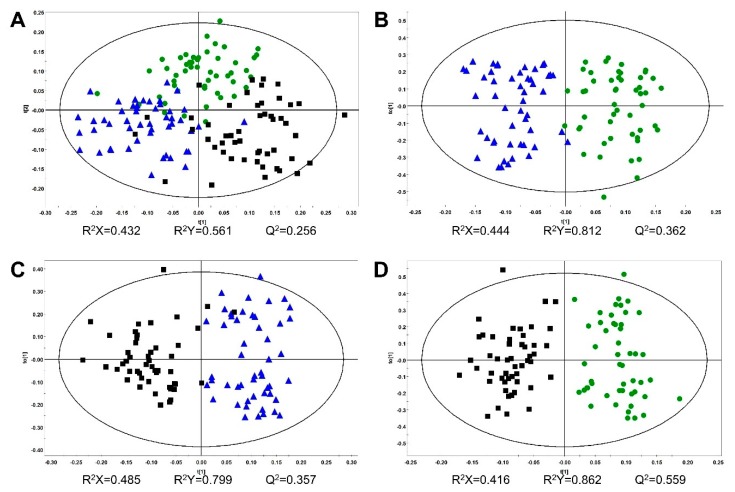
Orthogonal partial least squares discriminant analysis (OPLS-DA) score plots from NMR spectra: (**A**) 4-year-old (n = 50), 5-year-old (n = 50), and 6-year-old (n = 50) ginseng (discrimination rate: 85.33%); (**B**) 4- and 5-year-old ginseng (discrimination rate: 98%); (**C**) 5- and 6-year-old ginseng (discrimination rate: 97%); (**D**) 4- and 6-year-old ginseng (discrimination rate: 100%). ●, 4-year-old ginseng; ▲, 5-year-old ginseng; ■, 6-year-old ginseng.

Multivariate statistical analyses were conducted on Pareto-scaled NMR spectra of all samples. Score plots showed the clustering of different groups. PCA, PLS-DA, and OPLS-DA were conducted, however, the clustering patterns were not good in the PCA and PLS-DA models. In the OPLS-DA score plot, three groups were classified with an 85.33% discrimination rate (Figure 2A). The discrimination rates were obtained from a misclassification table in the SIMCA software. Comparisons of two groups were conducted to show clear clustering with a high discrimination rate close to 100% (Figure 2B–D). The OPLS model was a useful method of discriminating between the two groups [19].

### 2.2. UPLC–QTOF/MS-Based Metabolomics to Discriminate the Age of Ginseng

**Figure 3:**
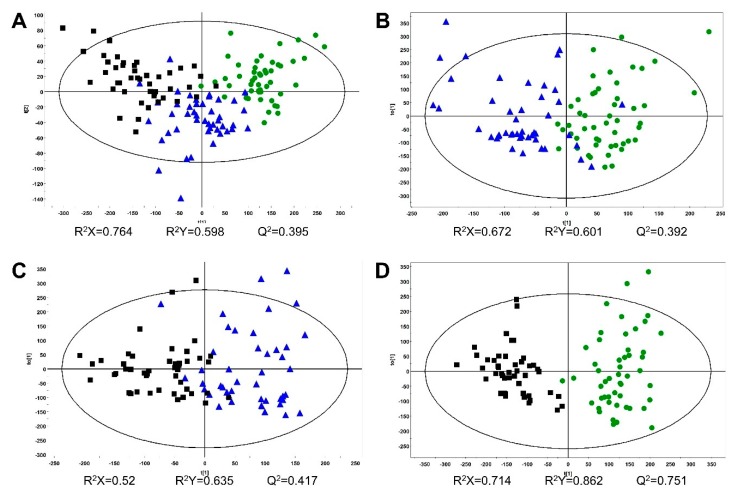
OPLS-DA score plots from metabolite profiling data of UPLC-QTOF/MS: (**A**) 4-year-old (*n* = 50), 5-year-old (*n* = 45), and 6-year-old (*n* = 46) ginseng (discrimination rate: 87.94%); (**B**) 4- and 5-year-old ginseng (discrimination rate: 92.63%); (**C**) 5- and 6-year-old ginseng (discrimination rate: 90.11%); (**D**) 4- and 6-year-old ginseng (discrimination rate: 98.96%). Outlier samples were excluded. ●, 4-year-old ginseng; ▲, 5-year-old ginseng; ■, 6-year-old ginseng.

**Figure 4:**
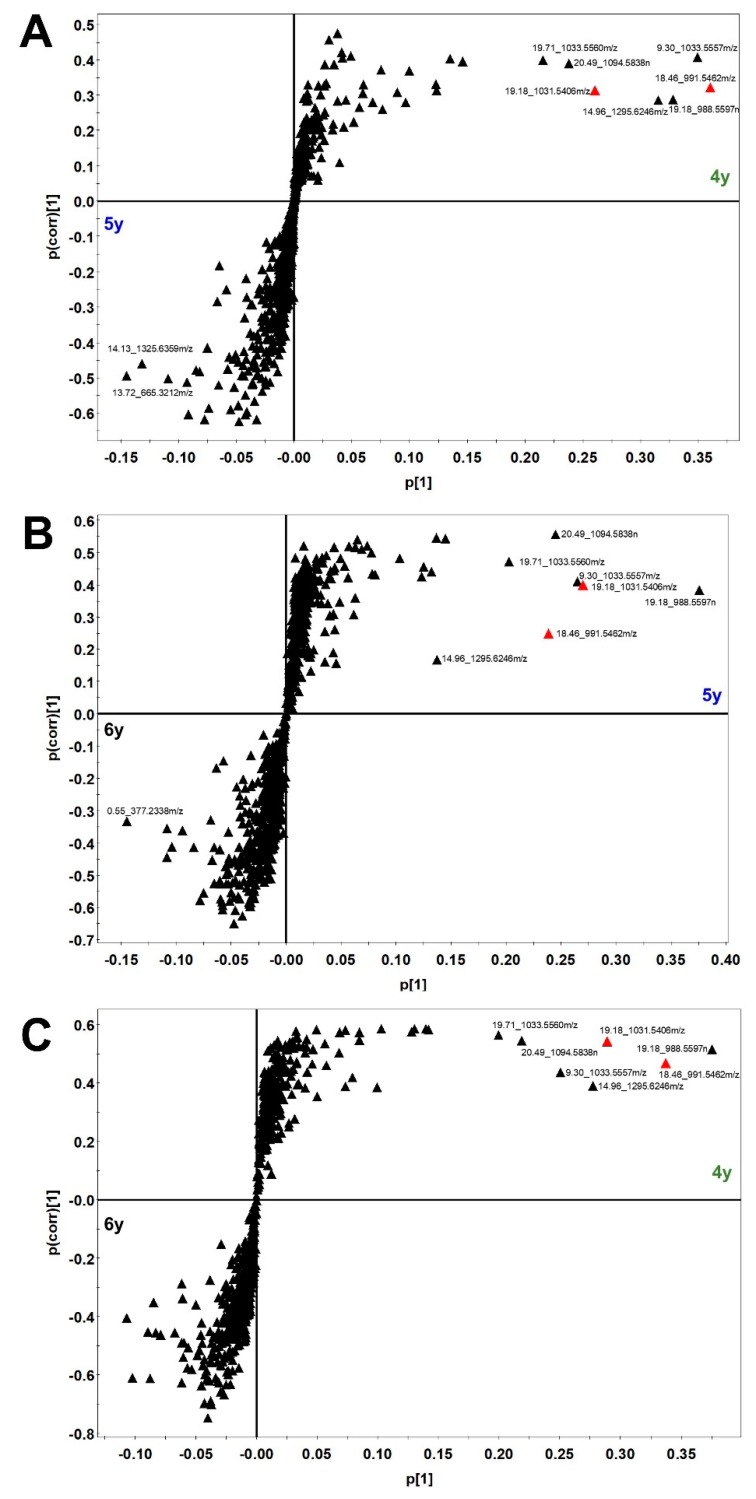
OPLS-DA loading S-plot from metabolite profiling data of UPLC-QTOF/MS: (**A**) 4- and 5-year-old ginseng; (**B**) 5- and 6-year-old ginseng; (**C**) 4- and 6-year-old ginseng.

A UPLC-QTOF/MS-based metabolomics approach was applied to discriminate ginseng of different ages. For this, the UPLC-QTOF/MS method developed for our previous study [20] was used to profile various metabolites in 4-, 5-, and 6-year-old ginseng samples. To extract the ginseng samples, 70% (*v*/*v*) methanol was used, and the extracts were then subjected to UPLC-QTOF/MS in negative ion mode. Figure S2 shows the representative base peak intensity (BPI) chromatograms of diverse metabolites taken from 4-, 5-, and 6-year-old ginseng samples. Depending on the age of the ginseng, the intensity of the several peaks varied, thus indicating that different growth periods/ages change the metabolic composition of ginseng. After the metabolite profiling of individual samples by UPLC-QTOF/MS, each set of data was processed using the UNIFI software. In total, 813 peaks, including unknown metabolites, were detected in the samples. Multivariate statistical analyses were conducted using metabolite profiling data of UPLC-QTOF/MS, which was Pareto-scaled. The OPLS-DA score plot showed classification of three groups with an 87.94% discrimination rate (Figure 3A). However, PCA and PLS-DA score plots showed poor classification of three groups. Comparisons of two groups using the OPLS-DA model showed a discrimination rate over 90% (Figure 3B–D). In order to confirm which variables contributed to age discrimination, S-plots were analyzed (Figure 4). The S-plot, which is one of the loading plots, is a visualization method that combines covariance (*x*-axis) and correlation loading profiles (*y*-axis) [21]. The farther away from the center, the more the variables contribute to clustering. Among these variables, ginsenoside Rd (retention time (RT), 18.46 min; *m/z*, 991.5462) and malonyl ginsenoside Rd (RT, 19.18 min; *m/z*, 1031.5406) were confirmed using an in-house library [20]. There was a high content of the two confirmed ginsenosides in the younger ginseng.

### 2.3. GC × GC–TOF/MS-Based Metabolomics to Discriminate the Age of Ginseng

**Figure 5:**
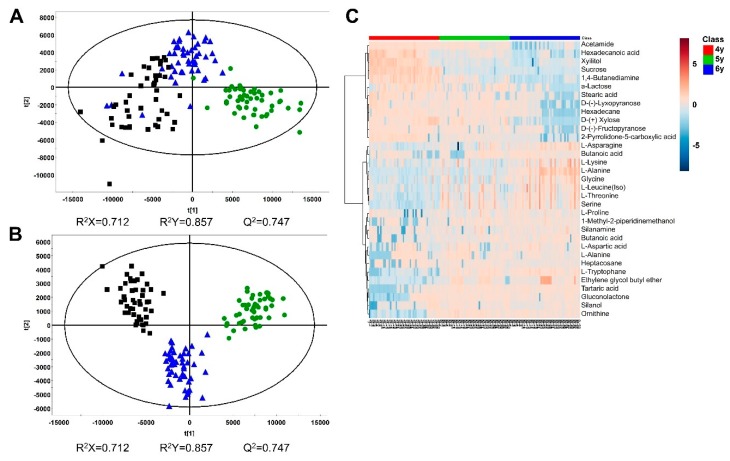
Multivariate statistical analyses of 4-year-old (*n* = 50), 5-year-old (*n* = 50), and 6-year-old (*n* = 50) ginseng profiling data obtained from GC × GC-TOF/MS. (**A**) PLS-DA score plot with a 98% discrimination rate. (**B**) OPLS-DA score plot with a 100% discrimination rate. ●, 4-year-old ginseng; ▲, 5-year-old ginseng; ■, 6-year-old ginseng. (**C**) Heatmap of 33 metabolites with a VIP score over 1.0 identified using the Agilent Fiehn GC/MS library and an in-house library.

Two-dimensional gas chromatography separation coupled to TOF mass spectrometry was optimized for the profiling of ginseng samples. After GC × GC-TOF/MS data processing using ChromaTOF software, a total of 186 signals were obtained. The TOF/MS was used to identify and quantify low-molecular-weight hydrophilic metabolites in ginseng samples. We were able to identify 33 compounds including 2 alcohols, 3 amines, 12 amino acids, 1 carbohydrate, 3 hydrocarbons, 6 organic acids, 5 sugars, and 1 sugar alcohol in theses samples (Figure 5C). The corresponding retention times agreed with our in-house libraries and the Fiehn Library [22] for standard chemicals.

Multivariate statistical analyses were conducted using all signals. PLS-DA and OPLS-DA score plots showed almost excellent classification of three groups with 98% and 100%, respectively, discrimination rates (Figure 5A,B). To confirm the more meaningful signals, VIP scores were calculated. Of the 186 variables, 43 showed significant VIP values (>1) as a result of PLS-DA. The main peaks were selected in the repeated components. After selection, a heatmap was generated using these 33 variables by MetaboAnalyst 4.0 (Figure 5C). In the heatmap, metabolic patterns were gradually changed by age. These patterns showed the discrimination of the three groups excellently. 

In order to check the metabolites that contributed to excellent age discrimination in most regions, receiver operating characteristic (ROC) analyses were conducted with all data and separate data in each region. ROC curves show sensitivity, specificity, and the area under the curve (AUC) value. The AUC value indicates how well the two groups are differentiated [23]. Generally, an AUC value of 0.9–1 is considered excellent, a value of 0.8–0.9 is good, a value of 0.7–0.8 is moderate, and a value below 0.7 is poor [24]. In our results, there were no matched metabolites in all regions. However, many metabolites excellently contributed to age discrimination (Tables S1 and S2).

## 3. Discussion

For effective discrimination of ginseng age, multiplex analytical methods were performed in this study. We applied three metabolomics-based approaches using HR-MAS NMR spectroscopy, UPLC-QTOF/MS, and GC × GC-TOF/MS to discriminate different ages of ginseng. HR-MAS NMR spectroscopy was used to study primary metabolites of ginseng powder with enhanced reproducibility [25]. High resolution can be obtained from samples not in a liquid state using the HR-MAS technique [26]. However, the disadvantage of NMR spectroscopy is its low sensitivity. To overcome this, chromatographic techniques combined with MS were used. UPLC-QTOF/MS is a powerful tool for metabolomics because it allows for separation of most compounds, ranging from hydrophilic to hydrophobic and including secondary metabolites. It has been widely used in nontarget metabolite studies because of its greater sensitivity and accurate mass measurement. GC-MS is a highly sensitive and comprehensive analytical tool for volatile and semivolatile organic compounds in mixture samples. A reference library of GC-MS has been established for many primary metabolites [27]. By using these multiple methods, we tried to obtain a complementary profile to discriminate age by checking the overall profile difference of primary and secondary metabolites in ginseng.

**Figure 6:**
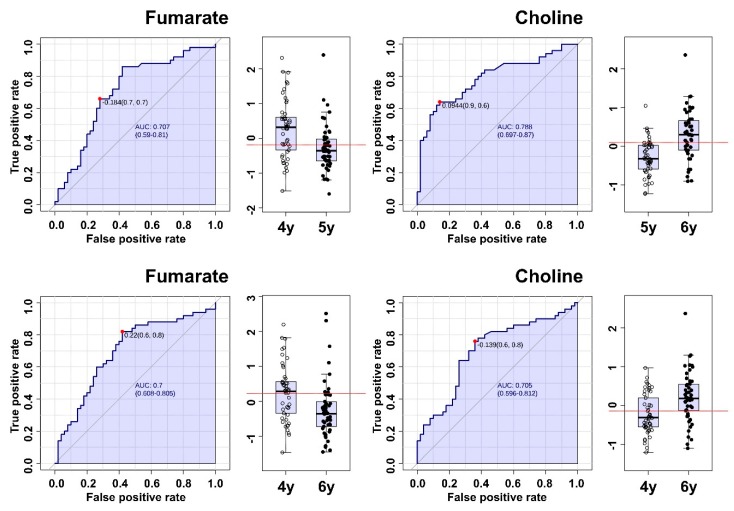
ROC curves and box plots of metabolites with AUC values of over 0.7 obtained from NMR spectroscopy to discriminate the age of ginseng.

The quantified primary metabolites from NMR spectra were analyzed to find the significant metabolites by the ROC curves using MetaboAnalyst 4.0. Fumarate (δ_H_ 6.51 (s)) had an AUC value of 0.707 for discrimination of 4- and 5-year-old ginseng. Choline ((δ_H_ 3.19 (s), 3.51 (dd), and 4.06 (ddd)) had an AUC value of 0.788 for discrimination of 5- and 6-year-old ginseng. Choline had AUC values over 0.7 in all regions. Moreover, in the Yeongju, AUC value was 0.97 with excellent prediction. For discrimination of 4- and 6-year-old ginseng, fumarate and choline had AUC values of 0.700 and 0.705, respectively (Figure 6).

**Figure 7:**
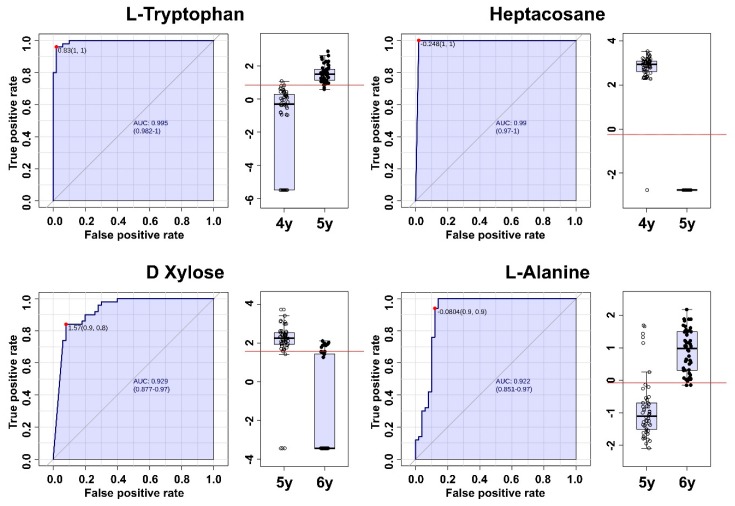
ROC curves and box plots of metabolites which ranked in the top two in each age comparison obtained from GC × GC-TOF/MS.

In order to find the metabolites that contribute to age prediction obtained from GC × GC-TOF/MS, ROC analyses were also conducted. Between 4- and 5-year-old ginseng, 19 metabolites showed excellent age prediction. In the ginseng between the ages of 5 and 6 years, only four metabolites showed excellent age prediction. The metabolites which can excellently predict age did not match in all regions. In most regions, 1H-indole-2,3-dione (except Goesan), 6-Methoxy-8-nitroquinoline (except Goesan), α-lactose (except Anseong), heptacosane (except Anseong), l-tryptophane (except Anseong), lyxose (except Anseong), and sucrose (except Anseong) were the matched metabolites for excellent prediction of age between 4- and 5-year-old ginseng. D-xylose (except Yeongju) was the matched metabolite in most regions for excellent prediction of age between 5- and 6-year-old ginseng (Figure 7). However, in the moderate predictable metabolites which had AUC values over 0.7 as meaningful metabolites for age discrimination, 16 metabolites between the 4- and 5-year-old ginseng and 18 metabolites between the 5- and 6-year-old ginseng were matched in all regions. In other words, since the differences in these matched metabolites are not in all of the regions, they are useful for age prediction.

**Figure 8:**
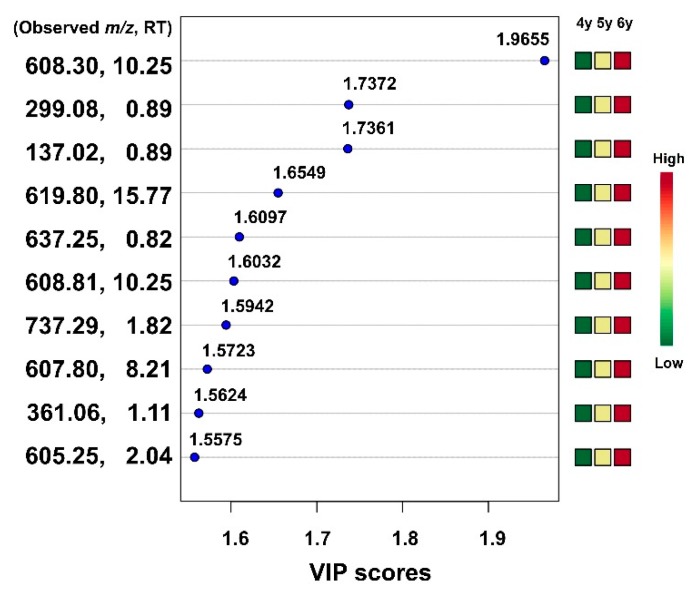
The top 10 variables ranked by VIP value from the PLS-DA analysis with data on UPLC–QTOF/MS of ginseng samples of different age.

In order to identify more influential variables for discriminating between ginseng samples of different ages in the UPLC-QTOF/MS data, the PLS-DA and VIP scores were repeatedly examined. The VIP scores indicate the metabolites’ importance in the PLS model [28]. VIP is the weighted sum of the squares of the PLS weight, and a value over 1.0 is typically used as a basis to identify the most important variables in a model [29]. Of the 813 variables, 301 showed significant VIP values (>1) as a result of PLS-DA. An additional PLS-DA was performed using the same 813 variables, which revealed 139 variables with a VIP value of more than 1. Finally, of the 813 variables, the 61 variables that played the greatest role in clear discrimination between ginseng samples of different ages were identified by repeating the PLS-DA three times (Figure 8). Among the metabolites analyzed from the 4-, 5-, and 6-year-old ginseng samples, 10 variables having significant VIP values (>1) played a role in discriminating between the metabolic profiles of ginseng samples of different ages. As these variables were not matched to the molecular information of ginsenosides, it is necessary to identify them by chromatographic isolation and structural identification using NMR spectroscopy. However, in this study, we only showed the RT and *m*/*z* values of each variable because it is difficult to perform identification of unknown variables. Finally, these results indicate that metabolomics can be a good tool for age discrimination of ginseng.

Using these three techniques allowed us to complement each’s pros and cons. This metabolic research contributes to the development of ginseng by facilitating its scientific management. First, HR-MAS NMR spectroscopy, which does not require any pretreatment such as extraction, was used to profile the primary metabolites in ginseng powder. Data can be easily and quickly obtained with easy measurement and analysis using NMR spectroscopy, but the number of metabolites to be identified is less than that provided by MS data. Second, UPLC-QTOF/MS was used to target the secondary metabolites contained in ginseng extract. Unlike NMR spectroscopy or GC×GC-TOF/MS, UPLC-QTOF/MS can identify secondary metabolites that reveal age differences. However, most of these are unknown variables. Third, GC × GC-TOF/MS was used to detect the comprehensive metabolites in ginseng extract. GC × GC-TOF/MS required a complex pretreatment process, but it was able to identify many metabolites and find meaningful metabolites that could certainly predict age using established databases. Although this study was limited to discriminating the age of ginseng using overall profiled data, these data can distinguish the age of ginseng excellently.

In future studies, it will be necessary to identify the unknown variables and to confirm the factors that change the variables related to age. If samples from various regions are analyzed in the future, further discussion of biologically active metabolites will be possible.

## 4. Materials and Methods 

### 4.1. Plant Materials

*Panax ginseng* was cultivated in five different regions, which are major production areas of ginseng in Korea: Yeongju in Gyeongsangbukk-do, Hoengseong in Gangwon-do, Jangsu in Jeollabuk-do, Anseong in Gyeongggi-do, and Goesan in Chungcheongbuk-do. It was cultivated according to the protocol of the “Ginseng GAP Standard Cultivation Guide” developed by the Rural Development Administration, Republic of Korea. Ginseng roots of 4-, 5-, and 6-year-old were harvested in 2017 (October). From each area, 10 samples of 4-year-old ginseng, 10 samples of 5-year- old ginseng, and 10 samples of 6-year-old ginseng were collected. A voucher specimen (NIHHS141010) was deposited at the herbarium of the Department of Herbal Crop Research, NIHHS, RDA, Eumseong, and the Republic of Korea.

### 4.2. Sample Preparation

In total, 50 samples of 4-year-old ginseng, 50 samples of 5-year-old ginseng, and 50 samples of 6-year-old ginseng were analyzed by HR-MAS NMR spectroscopy, UPLC–QTOF/MS, and GC × GC–TOF/MS. Each sample was washed and dried in a forced-air convection-drying oven at 40 °C for 48 h, and then weighed. The main roots were used in experiments after removing the lateral and fine roots. The roots were ground to 0.5 mm or less using a mixer (Hanil, Seoul, Korea) and the sub-samples were homogenized with a Retsch MM400 mixer mill (Retsch GmbH, Haan, Germany) for metabolic analyses.

### 4.3. NMR Experiments and Data Analysis

Deuterium oxide (D_2_O) and TSP-*d*_4_ were purchased from Sigma-Aldrich Co (St. Louis, MO). Each ginseng sample was weighed as 3 mg and transferred to an NMR nanotube (Agilent Technologies, Santa Clara, CA, USA). For the NMR analysis, 37 μL of D_2_O containing 2 mM TSP-*d*_4_ was added to each NMR nanotube. All the NMR spectra were acquired with a 600.167 MHz Agilent spectrometer equipped with a 4-mm gHX NanoProbe (Agilent Technologies). The spinning rate was set at 2000 Hz. The Carr–Purcell–Meiboom–Gill (CPMG) with the PRESAT pulse sequence was used in order to suppress high-molecular-mass compounds and water signal [30]. The spectra were obtained using 1.704 s of acquisition time, 1 s of relaxation delay, and 128 transients. The TSP-*d*_4_ peak at 0.00 ppm was used as a reference to calibrate the chemical shift [31].

All the spectra were phased and the baseline was corrected manually. Metabolite assignment and quantification of each sample were conducted using the Chenomx NMR Suite 7.1 professional (Chenomx Inc., Edmonton, AB, Canada) with 600 MHz library database of Chenomx. For the multivariate statistical analyses, each NMR spectrum was binned to 0.001 ppm of size from 0.83 to 6.8 ppm of area. Normalization was conducted with the total area. Alignments of the binned spectra were conducted using the Icoshift algorithm of MATLAB R2013b (MathWorks, Natick, MA, USA). After the alignment, multivariate statistical analyses were conducted using SIMCA-P+ 12.0 software (Umetrics, Umeå, Sweden) for PCA, PLS-DA, and OPLS-DA.

### 4.4. Reagents and the Extraction of Ginseng for UPLC–QTOF/MS Analysis

HPLC grade water, methanol, and acetonitrile were purchased from Fisher Scientific Korea (Seoul, Korea). HPLC grade formic acid was purchased from Fluka Chemie GmbH (Buchs, Switzerland). The fine ginseng powder was weighed as 100 mg, suspended in 1000 µL of 70% (*v*/*v*) methanol, and ultrasonically extracted at 50 °C for 30 min. Each extract was centrifuged at 13,500 rpm for 5 min. The extract was filtered through a syringe filter (0.22 µm) and injected directly into the UPLC system.

### 4.5. UPLC–QTOF/MS Experiments and Data Analysis

The UPLC analysis was performed using a Waters ACQUITY H-Class UPLC (Waters Corp., Milford, MA, USA) with a column of ACQUITY BEH C18 (1.7 µm, 2.1 mm × 100 mm,). The temperature of the column oven was set at 40 °C and the sample tray was set at 4 °C. The mobile phases were consisted with solvent A (water + 0.1% formic acid (*v*/*v*)) and solvent B (acetonitrile + 0.1% formic acid (*v*/*v*)).

Optimized UPLC elution conditions were as follows: 0–0.5 min, 15% B; 0.5–1 min, 20% B; 1–6 min, 20% B; 6–13 min, 30% B; 13–23 min, 35% B; 23–24 min, 38% B; 24–27 min, 60% B; 27–31 min, 90% B; 31–32 min, 15% B; 35 min, 15% B. The flow rate and the injection volume were 450 μL/min and 2 μL, respectively, for each run. Next, the MS analysis was performed using a Waters Xevo G2-S QTOF MS (Waters Corp.) operating in negative ion mode. Mass spectrometers were used to perform alternative high- and low-energy scans, otherwise known as the MS^E^ acquisition mode. The operating parameters were set as follows: capillary, 3.0 kV; source temperature, 120 °C; cone voltage, 40 V; cone gas flow, 30 L/h; desolvation temperature, 550 °C; and desolvation gas flow, 800 L/h. Accurate mass measurements were acquired using an automated calibration delivery system that contained an internal reference (Leucine-enkephalin, *m*/*z* 554.262 (ESI-)). Data were collected for *m*/*z* in the range between 100 and 2000. These methods were constructed and optimized in our lab [20].

All MS^E^ data were collected and processed using a UNIFI 1.8 (Waters Corp.). Data within the UNIFI 1.8 were passed through the apex peak detection and alignment processing algorithms. The intensity of each ion was normalized to the total ion count in order to generate a data matrix having an *m*/*z* value, RT, and normalized peak area. The charged species, salt adducts, and fragments were all automatically aligned and grouped. The normalized peak areas of aligned compounds were exported to the SIMCA-P+ 12.0 software (Umetrics, Umeå, Sweden) for the PCA, PLS-DA, and OPLS-DA.

### 4.6. Metabolite Extraction and Chemical Derivatization of Ginseng Samples for GC × GC–TOF/MS Analysis

Metabolites were extracted and derivatized as described previously [32]. The powdered ginseng samples (50 mg) were dissolved with 1,000 µL of pre-chilled chloroform:methanol:water (1:2.5:0.5) in order to extract the polar metabolites. Samples were then centrifuged at 13,000 rpm for 3 min at 4 °C and the resulting supernatant (100 µL) was transferred into a new tube and concentrated to dryness in a speedvac (CVE-3100, EYELA, Tokyo, Japan). Dried samples were mixed with 80 µL of MEOX reagent (methoxyamine hydrochloride in pyridine) and shaken (1200 rpm) for 60 min at 75 °C. This was followed by reaction with 100 μL of MSTFA (N-Methyl-N-trimethylsilyl trifluoroacetamide in pyridine) with 1% TMCS (chlorotrimethylsilane) at 60 °C for 1 h. The reaction products were cooled down to room temperature and injected directly for GC × GC–TOF/MS analysis. 

### 4.7. GC × GC–TOF/MS Experiments and Data Analysis

Polar metabolites were analyzed using an Agilent 7890 GC (Agilent Technologies Inc., Wilmington, DE, USA) with a dual-stage modulator, MPS2 auto-sampler (Gerstel Inc., Baltimore, MD, USA) and LECO Pegasus 4D (LECO Corp., St. Joseph, MI, USA), equipped with an electron ionization source and TOF analyzer. With a split ratio of 20:1, 1 µL of samples was analyzed. The first-dimension separation was carried out on a CP-SIL 8 CB fused-silica capillary column (30 m × 0.25 mm i.d. × 0.25 μm, Varian Inc., Palo Alto, CA, USA), while the second-dimension separation was done on an Rtx-200 capillary column (1.25 m × 0.25 mm i.d. × 0.25 μm, Restek Corp., Bellefonte, PA, USA). The injection temperature was set to 250 °C. Helium gas (1.0 mL/min) was used as a carrier gas and the head pressure was 90 kPa. The initial temperature of the first-dimension column was set at 50 °C and held for 4.5 min, then increased to 280 °C at a ramping rate of 10 °C/min and held for 4.5 min, whereas the secondary oven was set at a 5 °C offset above the primary oven. The modulator interface temperature was set at 15 °C above the secondary oven temperature. The modulation period was 2 s and the hot pulse was set at 0.8 s. The ion source and transfer line temperatures were set at 250 and 280 °C, respectively. The mass scanning range was 55–600 *m*/*z* at an acquisition rate of 100 spectra/s. The detector voltage was set at 1.7 kV, and the electron energy was set to 70 eV. For the data preprocessing, including baseline calculation, peak finding, deconvolution, and identification were conducted with ChromaTOF software (ver. 4.52, LECO). The method was as described above, as modified from previous studies [32,33]. Spectral similarity was searched in the Fiehn Library [21] and the in-house libraries for standard chemicals.

## Figures and Tables

**Table 1:** Fold changes of metabolites detected from NMR spectra of powdered ginseng.

(5 Years Old)/(4 Years Old)		(6 Years Old)/(5 Years Old)	
Fumarate **	0.7182	Glucose **	0.4695
Valine *	0.8432	Glycerophosphocholine	0.7069
4-Aminobutyrate *	0.8604	Malate **	0.7503
Sucrose	0.8878	Aspartate **	0.8079
Isoleucine	0.8908	Glutamate	0.8772
Leucine *	0.9161	Proline	0.9321
Glutamine	0.9200	Inositol	0.9888
Phenylalanine	0.9269	Leucine	1.0034
Choline	0.9316	Threonine	1.0220
Arginine	0.9591	Isoleucine	1.0240
Alanine	0.9794	Phosphocholine	1.0241
Proline	0.9832	Ethanolamine	1.0373
Ethanolamine	0.9927	Phenylalanine	1.0429
Phosphocholine	1.0485	Arginine	1.0586
Malate	1.0586	Asparagine	1.0754
Threonine	1.0891	Glutamine	1.0774
Tyrosine	1.0971	Fumarate	1.0892
Aspartate *	1.1649	Sucrose	1.1080
Glycerophosphocholine	1.2048	Valine *	1.1175
Inositol	1.2074	Tyrosine *	1.1258
Glutamate *	1.2757	Alanine	1.1412
Asparagine **	1.3251	4-Aminobutyrate	1.1817
Glucose	1.4969	Choline **	1.3195

The symbols * and ** are used to indicate statistical significance with *p* < 0.05 and *p* < 0.01, respectively.

## Supplementary Materials

The following are available online at https://www.mdpi.com/1420-3049/24/13/2381/s1, Figure S1: Representative ^1^H-NMR spectra from 600 MHz NMR spectrometer of (A) 4-years old, (B) 5-years old, and (C) 6-years old ginseng samples, Figure S2: Representative UPLC–QTOF/MS base peak intensity (BPI) chromatograms of (A) 4-years old, (B) 5-years old, and (C) 6-years old ginseng samples, Figure S3: Representative GC×GC–TOF/MS total ion chromatograms (TIC) of (A) 4-years old, (B) 5-years old, and (C) 6-years old ginseng samples, Table S1: AUC values between 4- and 5-years old ginseng, Table S2: AUC values between 5- and 6-years old ginseng.

## Abbreviations

ESI	Electrospray ionization
MS	Mass spectrometry
RT	Retention time
