# Supplementary material for: Metabolomics for Age Discrimination of Ginseng Using a Multiplex Approach to HR-MAS NMR Spectroscopy, UPLC–QTOF/MS, and GC × GC–TOF/MS
Source: Molecules. 2019 Jun 27;24(13):2381. doi: 10.3390/molecules24132381 (PMC6651322; doi:10.3390/molecules24132381)
Supplement: Supplementary file 1 [file molecules-24-02381-s001.zip › Table S1.docx]

Table S1. AUC values between 4- and 5-years old ginseng

|  | **AUC** | **Pval** | **FC** | **Sampling Area** |
| --- | --- | --- | --- | --- |
| Shikimic acid | 0.93 | 4.01747E-05 | -0.85778 | Yeongju |
| 1,3-Dioxolane | 0.91 | 0.000541458 | 0.709089 | Yeongju |
| 1,3-Dioxolane | 0.9 | 0.000730432 | 0.827902 | Goesan |
| 1,5-Anhydro-D-sorbitol | 0.93 | 0.066130816 | 2.479572 | Yeongju |
| 1,5-Anhydro-D-sorbitol | 0.92 | 0.004285202 | 1.257638 | Hoengseong |
| 3-Methyl-1H-1,2,4-triazole | 0.92 | 0.021131547 | -6.7273 | Hoengseong |
| 2-Cyclohexyl-1H-indole | 0.96 | 0.00483897 | -0.39433 | Anseong |
| 1H-Indole-2,3-dione | 1 | 0.020911492 | 4.476212 | Yeongju |
| 1H-Indole-2,3-dione | 0.9 | 0.960281175 | -0.34445 | Hoengseong |
| 1H-Indole-2,3-dione | 0.95 | 0.08749761 | 2.00879 | Jangsu |
| 1H-Indole-2,3-dione | 1 | 0.070281018 | -2.8135 | Anseong |
| 1-Iodo-2-methylundecane | 0.9 | 0.000212415 | 0.689064 | Anseong |
| 1-Methyl-2-piperidinemethanol | 0.97 | 0.002790952 | -6.91799 | Jangsu |
| 1-Methyl-2-piperidinemethanol | 0.93 | 0.125345622 | -1.99303 | Goesan |
| 2-Butenedioic acid | 0.9 | 0.001322894 | -1.10966 | Hoengseong |
| 2-Butenedioic acid | 0.96 | 4.79361E-05 | -1.01749 | Jangsu |
| 2-Pentenoic acid | 1 | 6.93535E-05 | -0.52832 | Goesan |
| 2-Pyrrolidone-5-carboxylic acid | 0.94 | 0.000126651 | 1.054158 | Yeongju |
| 2-Pyrrolidone-5-carboxylic acid | 0.95 | 0.000100316 | 0.964235 | Hoengseong |
| 2-Pyrrolidone-5-carboxylic acid | 0.91 | 0.00018709 | 1.239078 | Jangsu |
| (3E,5Z)-6-tert-butyl-2,2,9,9-tetramethyl-deca-3,5-dien-7-yne | 0.94 | 0.000650785 | 0.581253 | Yeongju |
| (3E,5Z)-6-tert-butyl-2,2,9,9-tetramethyl-deca-3,5-dien-7-yne | 0.94 | 0.000291417 | 0.496298 | Hoengseong |
| (3E,5Z)-6-tert-butyl-2,2,9,9-tetramethyl-deca-3,5-dien-7-yne | 0.9 | 0.000845321 | 0.521298 | Jangsu |
| 3,6,9,12-Tetraoxa-2,13-disilatetradecane | 0.93 | 0.000698181 | 0.360436 | Yeongju |
| 3,6,9,12-Tetraoxa-2,13-disilatetradecane | 0.95 | 0.004879597 | -0.34311 | Anseong |
| 4-1,1-Dimethylpropylphenol | 0.98 | 0.001049604 | -0.38701 | Anseong |
| 6,8-Dimethyl-7-phenyl-1,3,8-triazaspiro4.5decan-2,4-dithione | 0.96 | 4.22509E-05 | 0.408244 | Yeongju |
| 6,8-Dimethyl-7-phenyl-1,3,8-triazaspiro4.5decan-2,4-dithione | 0.98 | 5.83824E-05 | 0.455977 | Jangsu |
| 6,8-Dimethyl-7-phenyl-1,3,8-triazaspiro4.5decan-2,4-dithione | 0.97 | 0.000515566 | -0.44682 | Anseong |
| 6-Methoxy-3-methyl-5-isopropoxy-8-nitroquinoline | 1 | 2.61816E-08 | 1.08897 | Yeongju |
| 6-Methoxy-3-methyl-5-isopropoxy-8-nitroquinoline | 0.9 | 0.796696669 | -0.08877 | Hoengseong |
| 6-Methoxy-3-methyl-5-isopropoxy-8-nitroquinoline | 1 | 7.97692E-12 | 1.23829 | Jangsu |
| 6-Methoxy-3-methyl-5-isopropoxy-8-nitroquinoline | 1 | 0.06002718 | -2.98711 | Anseong |
| 7,9-Di-tert-butyl-1-oxaspiro4,5deca-6,9-diene-2,8-dione | 1 | 4.69256E-10 | 1.19761 | Jangsu |
| α-Lactose | 0.93 | 0.000290573 | -0.60227 | Yeongju |
| α-Lactose | 0.92 | 0.00058967 | -0.46967 | Hoengseong |
| α-Lactose | 0.95 | 3.56307E-05 | -0.74998 | Jangsu |
| α-Lactose | 1 | 7.00463E-07 | -0.63341 | Goesan |
| Butanoic acid | 0.92 | 0.000108485 | -10.2679 | Goesan |
| Cinnamic acid | 1 | 0.014843735 | 4.455332 | Yeongju |
| Cinnamic acid | 1 | 0.00369181 | 3.982901 | Hoengseong |
| Cinnamic acid | 1 | 4.33345E-10 | 2.195448 | Jangsu |
| Citrulline | 0.9 | 0.035610039 | -2.8932 | Anseong |
| D-Raffinose | 0.93 | 0.000733514 | -1.25428 | Yeongju |
| D-Raffinose | 0.92 | 0.001925831 | -0.18041 | Goesan |
| D-Trehalose | 0.93 | 0.000103062 | 1.399298 | Goesan |
| D-Xylose | 0.94 | 0.014001188 | -3.46616 | Hoengseong |
| D-Xylose | 1 | 2.21749E-21 | 14.50162 | Anseong |
| D-Xylose | 0.91 | 0.021774824 | 6.329126 | Goesan |
| D-Fructopyranose | 0.96 | 8.36434E-05 | 1.638262 | Goesan |
| D-Glucopyranose | 0.95 | 2.84778E-05 | 1.085809 | Goesan |
| D-Lyxofuranose | 0.94 | 0.115826204 | 2.541302 | Anseong |
| D-Lyxopyranose | 0.95 | 0.000481497 | 8.25337 | Anseong |
| D-Talofuranose | 0.95 | 2.82219E-05 | 1.8103 | Goesan |
| D-Talopyranose | 0.96 | 9.32378E-06 | 0.924384 | Goesan |
| D-Talopyranose | 0.96 | 0.000370591 | -0.49023 | Yeongju |
| D-Talopyranose | 1 | 0.038075045 | 2.502246 | Anseong |
| D-Valine | 0.9 | 0.019853733 | -0.55707 | Jangsu |
| Ethyl 2,3,4,6-tetrakis-O-trimethylsilyl-D-glucopyranoside | 0.92 | 0.002681671 | -0.75742 | Yeongju |
| Ethyl 2,3,4,6-tetrakis-O-trimethylsilyl-D-glucopyranoside | 0.95 | 0.000140779 | -0.48676 | Goesan |
| Ethylene glycol butyl ether | 1 | 6.6096E-07 | -3.79079 | Yeongju |
| Ethylene glycol butyl ether | 1 | 2.21239E-06 | -5.61312 | Hoengseong |
| Ethylene glycol butyl ether | 1 | 1.26231E-06 | -3.10554 | Jangsu |
| Gluconolactone | 1 | 8.68998E-06 | -8.73427 | Yeongju |
| Gluconolactone | 1 | 7.23232E-08 | -9.70218 | Hoengseong |
| Gluconolactone | 0.94 | 1.72337E-07 | -5.65038 | Jangsu |
| Glutamic acid | 0.96 | 0.000243682 | -0.80656 | Jangsu |
| Glycine | 0.9 | 0.001830403 | -0.84557 | Yeongju |
| Glycine | 0.98 | 6.92354E-05 | -0.572 | Jangsu |
| Heptacosane | 1 | 7.71455E-22 | 16.28671 | Yeongju |
| Heptacosane | 1 | 1.37138E-21 | 14.73956 | Hoengseong |
| Heptacosane | 1 | 8.85844E-25 | 16.82995 | Jangsu |
| Heptacosane | 1 | 1.09538E-21 | 16.11697 | Goesan |
| Hexadecane | 0.9 | 0.574198401 | -0.95163 | Yeongju |
| Hexadecane | 1 | 7.08463E-09 | 13.58664 | Anseong |
| Hexamethyldisilazane | 1 | 1.91044E-07 | 0.983712 | Yeongju |
| Hexamethyldisilazane | 0.98 | 0.000132165 | 0.53493 | Hoengseong |
| Hexamethyldisilazane | 1 | 4.68893E-08 | 0.861443 | Jangsu |
| Ibuprofen | 1 | 5.48891E-06 | 0.700456 | Yeongju |
| Ibuprofen | 1 | 4.20852E-07 | 0.620169 | Hoengseong |
| Ibuprofen | 1 | 1.2702E-07 | 0.620462 | Jangsu |
| L-Alanine | 0.94 | 0.006488245 | -1.36781 | Yeongju |
| L-Alanine | 0.94 | 0.002615767 | -1.13068 | Jangsu |
| L-Alanine | 0.9 | 2.8445E-05 | -2.4084 | Anseong |
| L-Asparagine | 0.97 | 0.005770859 | 7.045156 | Yeongju |
| L-Asparagine | 0.95 | 0.024974026 | -3.28553 | Anseong |
| L-Aspartic acid | 1 | 1.88667E-07 | 1.446087 | Yeongju |
| L-Aspartic acid | 1 | 1.87823E-05 | 1.189229 | Jangsu |
| L-Aspartic acid | 0.97 | 8.18519E-05 | -0.83586 | Anseong |
| L-Leucine | 1 | 0.008097388 | -3.64754 | Yeongju |
| L-Leucine | 0.9 | 0.394397109 | -0.70319 | Hoengseong |
| L-Lysine | 0.93 | 0.001118447 | -1.20176 | Yeongju |
| L-Ornithine monohydrochloride | 0.905 | 0.222296077 | -2.4832 | Yeongju |
| L-Ornithine monohydrochloride | 1 | 2.41352E-09 | -2.48679 | Hoengseong |
| L-Ornithine monohydrochloride | 0.95 | 0.094852694 | -2.48868 | Goesan |
| L-Proline | 0.91 | 0.052808086 | 3.860936 | Jangsu |
| L-Serine | 1 | 3.12117E-06 | -1.5634 | Yeongju |
| L-Serine | 0.96 | 0.005646937 | -0.95536 | Hoengseong |
| L-Serine | 1 | 3.83492E-06 | -1.41271 | Jangsu |
| L-Tartaric acid | 1 | 1.46833E-24 | -13.9449 | Yeongju |
| L-Tartaric acid | 1 | 4.5441E-25 | -12.3789 | Hoengseong |
| L-Tartaric acid | 1 | 0.006632223 | -5.70512 | Jangsu |
| L-Threonine | 0.96 | 0.000604281 | -1.22565 | Yeongju |
| L-Threonine | 1 | 1.24933E-05 | -1.2235 | Jangsu |
| L-Tryptophane | 1 | 3.51023E-08 | -14.4449 | Yeongju |
| L-Tryptophane | 1 | 4.13451E-05 | -5.38072 | Hoengseong |
| L-Tryptophane | 1 | 2.99885E-06 | -2.59151 | Jangsu |
| L-Tryptophane | 0.97 | 0.003620912 | -7.14067 | Goesan |
| Lyxose | 0.95 | 2.46977E-05 | 1.024716 | Yeongju |
| Lyxose | 1 | 9.30317E-05 | 1.248564 | Hoengseong |
| Lyxose | 0.98 | 0.000328295 | 1.509102 | Jangsu |
| Lyxose | 1 | 0.018783194 | 3.582236 | Goesan |
| N,N-Diethyl-1,1,1-trimethylsilylamine | 0.94 | 0.000431848 | 0.478667 | Yeongju |
| Niacinamide | 0.97 | 9.84608E-05 | -0.44423 | Goesan |
| Octaethylene glycol monododecyl ether | 0.95 | 0.000486167 | 0.536263 | Hoengseong |
| Octaethylene glycol monododecyl ether | 0.92 | 0.001645643 | 0.39419 | Jangsu |
| Octane | 0.96 | 1.38151E-05 | 0.856876 | Hoengseong |
| Octane | 0.99 | 6.98985E-07 | 0.695895 | Jangsu |
| Ornithine | 1 | 9.07347E-05 | -3.77829 | Yeongju |
| Ornithine | 1 | 4.46319E-08 | -2.6211 | Hoengseong |
| Ornithine | 1 | 1.0863E-09 | -3.21096 | Jangsu |
| Oxalic acid | 0.98 | 3.85166E-06 | 0.945812 | Jangsu |
| Palmitic acid | 1 | 0.025539183 | 3.688544 | Anseong |
| Probucol | 1 | 0.009204125 | 4.927421 | Anseong |
| Silanamine | 1 | 4.12696E-07 | -0.95442 | Anseong |
| Silanol | 1 | 4.12167E-18 | 2.522273 | Yeongju |
| Silanol | 1 | 0.000747026 | 3.713016 | Hoengseong |
| Silanol | 1 | 5.07919E-12 | 2.654988 | Jangsu |
| Sitosterol | 0.94 | 0.000154455 | -0.67774 | Goesan |
| Stearic acid | 1 | 8.052E-06 | 0.614609 | Yeongju |
| Stearic acid | 0.94 | 0.000301881 | 0.413481 | Jangsu |
| Sucrose | 0.98 | 0.003983117 | 5.278519 | Yeongju |
| Sucrose | 1 | 2.06257E-12 | 4.267635 | Hoengseong |
| Sucrose | 1 | 3.78546E-13 | 5.140663 | Jangsu |
| Sucrose | 1 | 8.35509E-09 | 5.187796 | Goesan |
| Tetradecane | 1 | 8.81164E-09 | -1.39805 | Jangsu |
| Triethylamine | 0.96 | 3.23391E-05 | 0.810292 | Yeongju |
| Triethylamine | 0.99 | 3.8853E-06 | 0.794088 | Jangsu |
| Xyilitol | 1 | 2.79005E-08 | 2.718984 | Yeongju |
| Xyilitol | 1 | 8.6068E-12 | 2.543427 | Hoengseong |
| Xyilitol | 1 | 3.97235E-08 | 1.937238 | Jangsu |
