# Supplementary material for: Metabolomics for Age Discrimination of Ginseng Using a Multiplex Approach to HR-MAS NMR Spectroscopy, UPLC–QTOF/MS, and GC × GC–TOF/MS
Source: Molecules. 2019 Jun 27;24(13):2381. doi: 10.3390/molecules24132381 (PMC6651322; doi:10.3390/molecules24132381)
Supplement: Supplementary file 1 [file molecules-24-02381-s001.zip › Table S2.docx]

Table S2. AUC values between 5- and 6-years old ginseng

|  | **AUC** | **Pval** | **FC** | **Sampling Area** |
| --- | --- | --- | --- | --- |
| 1,4-Butanediamine | 0.98 | 0.00000156 | 1.546615 | Yeongju |
| 1,4-Butanediamine | 1 | 5.87E-08 | 1.715978 | Hoengseong |
| 1H-1,2,4-Triazole | 0.92 | 0.060210038 | 3.992351 | Jangsu |
| 1H-Indole | 0.96 | 0.00483897 | -0.39433 | Anseong |
| 1H-Indole-2,3-dione | 1 | 0.070281018 | -2.8135 | Anseong |
| 1-Iodo-2-methylundecane | 0.9 | 0.000212415 | 0.689064 | Anseong |
| 1-Iodo-2-methylundecane | 0.95 | 0.000670218 | 0.608612 | Goesan |
| 2-Butenedioic acid | 0.99 | 0.0000398 | -0.41812 | Yeongju |
| 2-Butenedioic acid | 0.99 | 0.000000892 | -1.00675 | Hoengseong |
| 2-Butenedioic acid | 0.98 | 0.0000143 | -0.45715 | Jangsu |
| 2-Pyrrolidone-5-carboxylic acid | 0.9 | 0.003764621 | 0.587324 | Yeongju |
| 2-Pyrrolidone-5-carboxylic acid | 0.96 | 0.010926115 | 7.387809 | Jangsu |
| 2-Pyrrolidone-5-carboxylic acid | 0.98 | 0.065589434 | 3.96502 | Goesan |
| 3,6,9,12-Tetraoxa-2,13-disilatetradecane | 0.95 | 0.004879597 | -0.34311 | Anseong |
| 4-1,1-Dimethylpropylphenol | 0.98 | 0.001049604 | -0.38701 | Anseong |
| 6,8-Dimethyl-7-phenyl-1,3,8-triazaspiro4.5decan-2,4-dithione | 0.97 | 0.000515566 | -0.44682 | Anseong |
| 6-Methoxy-3-methyl-5-isopropoxy-8-nitroquinoline | 1 | 0.06002718 | -2.98711 | Anseong |
| 7,9-Di-tert-butyl-1-oxaspiro4,5deca-6,9-diene-2,8-dione | 0.98 | 0.017647456 | 2.753471 | Hoengseong |
| Acetamide | 1 | 0.00040503 | 9.390392 | Yeongju |
| Acetamide | 1 | 0.000445068 | 9.116907 | Hoengseong |
| Acetamide | 0.92 | 0.013569763 | 5.962736 | Jangsu |
| α-Lactose | 0.95 | 4.81E-08 | 13.67426 | Goesan |
| Alanine | 0.94 | 0.000118962 | -1.42134 | Jangsu |
| Butanoic acid | 0.94 | 0.00000679 | -11.9904 | Hoengseong |
| Citrulline | 0.9 | 0.035610039 | -2.8932 | Anseong |
| D-Raffinose | 0.96 | 0.0000919 | 0.975272 | Yeongju |
| D-Raffinose | 0.91 | 0.000472089 | 0.835627 | Hoengseong |
| D-Raffinose | 0.95 | 0.000529003 | 0.589976 | Goesan |
| D-Xylose | 0.98 | 0.002142718 | 8.277513 | Hoengseong |
| D-Xylose | 0.96 | 0.0000514 | 11.75287 | Jangsu |
| D-Xylose | 1 | 2.22E-21 | 14.50162 | Anseong |
| D-Xylose | 0.9 | 0.0000156 | 14.4184 | Goesan |
| D-Fructopyranose | 0.93 | 0.148637746 | 2.968131 | Yeongju |
| D-Lyxofuranose | 0.94 | 0.115826204 | 2.541302 | Anseong |
| D-Lyxofuranose | 0.9 | 0.150118078 | 2.563918 | Goesan |
| D-Lyxopyranose | 0.94 | 0.000244307 | 9.178605 | Jangsu |
| D-Lyxopyranose | 0.95 | 0.000481497 | 8.25337 | Anseong |
| D-Lyxopyranose | 1 | 0.000244894 | 8.725107 | Goesan |
| D-Talofuranose | 0.92 | 0.036749057 | 6.415818 | Hoengseong |
| D-Talopyranose | 0.92 | 0.000829648 | 1.344386 | Jangsu |
| D-Talopyranose | 1 | 0.038075045 | 2.502246 | Anseong |
| D-Talopyranose | 1 | 0.0000137 | 1.392889 | Goesan |
| D-Valine | 0.96 | 0.000043 | -1.12331 | Goesan |
| Ethyl 2,3,4,6-tetrakis-O-trimethylsilyl-D-glucopyranoside | 0.94 | 0.000506916 | -0.72245 | Hoengseong |
| Ethylbistrimethylsilylamine | 0.93 | 0.000374422 | 0.090224 | Goesan |
| Ethylene glycol butyl ether | 0.96 | 0.0000141 | -5.79846 | Jangsu |
| Ethylene glycol butyl ether | 0.99 | 0.018733119 | -6.1544 | Goesan |
| Gluconolactone | 0.92 | 0.002421272 | -0.68839 | Hoengseong |
| Gluconolactone | 0.91 | 0.001049556 | -0.83806 | Jangsu |
| Glutamic acid | 0.98 | 0.0000154 | -0.74559 | Hoengseong |
| N-acetylglycine | 0.94 | 0.000593627 | 0.066006 | Goesan |
| Glycine | 0.99 | 0.0000444 | -1.07933 | Goesan |
| Heptacosane | 0.9 | 0.129419123 | 3.601447 | Hoengseong |
| Heptacosane | 0.92 | 0.002296589 | 0.276964 | Jangsu |
| Hexadecane | 1 | 7.08E-09 | 13.58664 | Anseong |
| Hexadecane | 1 | 1.03E-08 | 14.81656 | Goesan |
| Hexadecanoic acid | 0.9 | 0.000996348 | 0.713198 | Hoengseong |
| Hexadecanoic acid | 1 | 6.59E-09 | 1.599675 | Anseong |
| Hexamethyldisilazane | 0.99 | 0.000000735 | 0.46704 | Hoengseong |
| L-Alanine | 0.95 | 0.00000464 | -2.51763 | Jangsu |
| L-Alanine | 0.9 | 0.0000284 | -2.4084 | Anseong |
| L-Alanine | 1 | 6.17E-09 | -2.65658 | Goesan |
| L-Asparagine | 1 | 7.74E-34 | -12.5374 | Yeongju |
| L-Asparagine | 1 | 4.93E-31 | -12.1357 | Hoengseong |
| L-Asparagine | 0.94 | 0.000379707 | -0.53785 | Anseong |
| L-Aspartic acid | 0.91 | 0.000809394 | 0.999071 | Hoengseong |
| L-Aspartic acid | 0.97 | 0.0000819 | -0.83586 | Anseong |
| Levoglucosan | 0.92 | 0.083731015 | 4.276096 | Jangsu |
| L-LeucineIso | 0.97 | 0.0000564 | -1.32062 | Goesan |
| L--Tartaric acid | 0.96 | 0.0000552 | -0.50787 | Jangsu |
| L-Threonine | 0.94 | 0.000323722 | -0.92011 | Goesan |
| L-Tyrosine | 0.91 | 0.000760664 | -1.13915 | Goesan |
| Lyxose | 0.9 | 0.544778027 | -0.90517 | Yeongju |
| N,N-Diethyl-1,1,1-trimethylsilylamine | 0.94 | 0.009992251 | 0.178237 | Goesan |
| N-Acetyl-L-Lysine | 0.91 | 0.047239904 | -3.8645 | Jangsu |
| N-Acetyl-L-Lysine | 0.9 | 0.052689506 | -3.07758 | Goesan |
| Oxalic acid | 0.95 | 0.0000609 | 0.864598 | Jangsu |
| Oxalic acid | 0.99 | 0.00000422 | 0.701719 | Goesan |
| Palmitic acid | 0.99 | 0.126884878 | 1.094142 | Jangsu |
| Palmitic acid | 0.97 | 0.0000108 | 0.229098 | Goesan |
| Probucol | 1 | 0.009204125 | 4.927421 | Anseong |
| Ribonic acid | 0.98 | 0.0000787 | -0.91378 | Hoengseong |
| Ribonic acid | 0.96 | 0.003761848 | -2.26391 | Jangsu |
| Silanamine | 1 | 0.000000413 | -0.95442 | Anseong |
| Sitosterol | 0.92 | 0.000842621 | -0.23747 | Yeongju |
| Stearic acid | 0.92 | 0.0000901 | 0.792823 | Jangsu |
| Stearic acid | 0.96 | 0.00000224 | 0.535372 | Anseong |
| Stearic acid | 0.99 | 0.00000396 | 0.496203 | Goesan |
| Succinic acid | 0.99 | 0.00000122 | -1.67274 | Hoengseong |
| Succinic acid | 0.92 | 0.001536419 | -0.70634 | Jangsu |
| Tetradecane | 0.9 | 0.0001216 | -1.48797 | Hoengseong |
| trans-2-Phenylcyclopropylamine | 1 | 0.0000165 | -0.62471 | Anseong |
| Triethylamine | 1 | 9.57E-09 | 0.600535 | Hoengseong |
| Triethylamine | 0.98 | 0.00000441 | 0.477266 | Goesan |
| Undecane | 0.9 | 0.00066802 | -0.40416 | Hoengseong |
| Xyilitol | 0.93 | 0.053507751 | -2.36584 | Hoengseong |
| Xyilitol | 0.97 | 0.045494296 | -2.42183 | Jangsu |
