# Supplementary figures and images for: Metabolomics for Age Discrimination of Ginseng Using a Multiplex Approach to HR-MAS NMR Spectroscopy, UPLC–QTOF/MS, and GC × GC–TOF/MS
Source: Molecules. 2019 Jun 27;24(13):2381. doi: 10.3390/molecules24132381 (PMC6651322; doi:10.3390/molecules24132381)

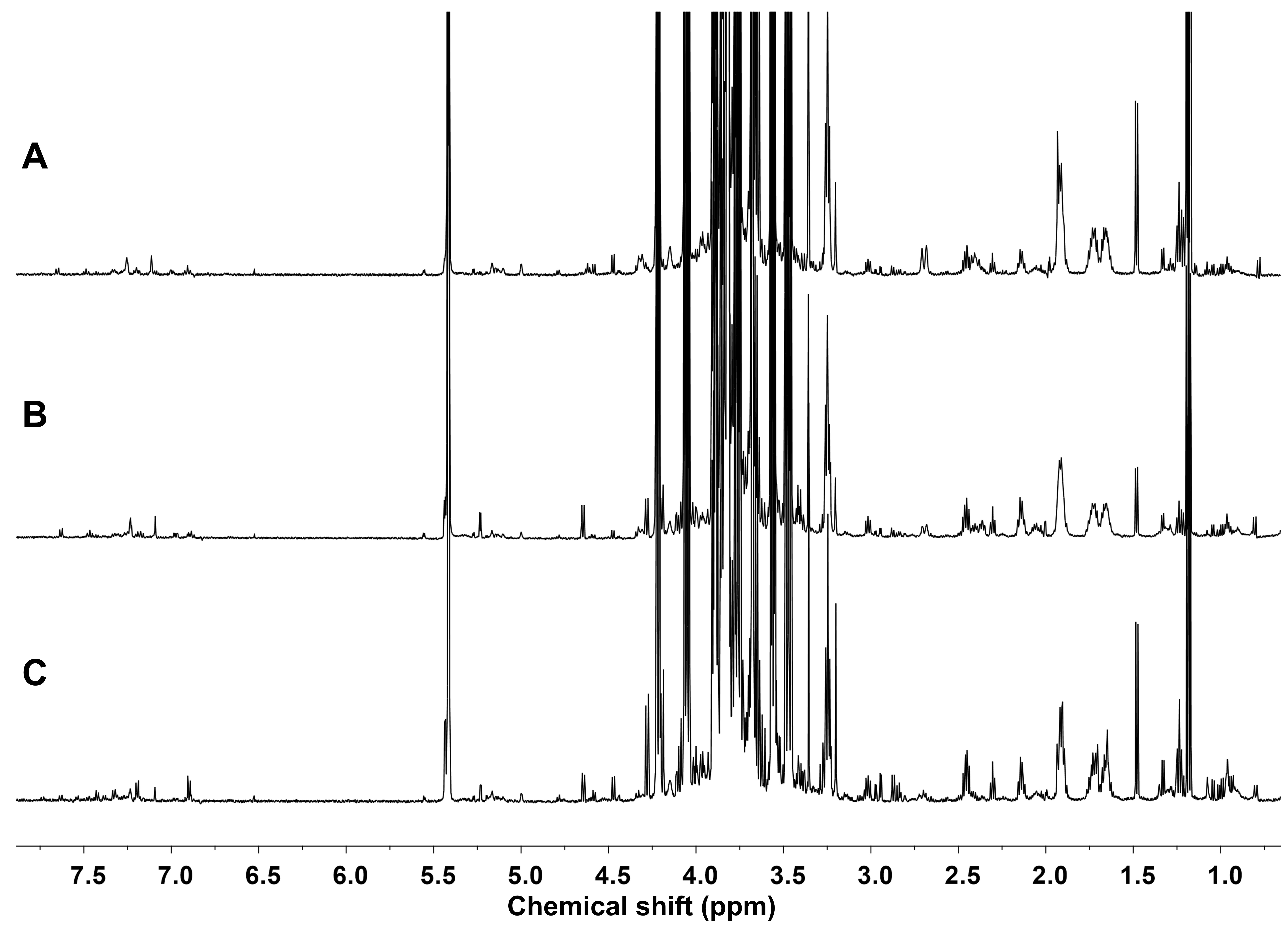

Supplement: Supplementary file 1 [file molecules-24-02381-s001.zip › FigureS1.jpg]

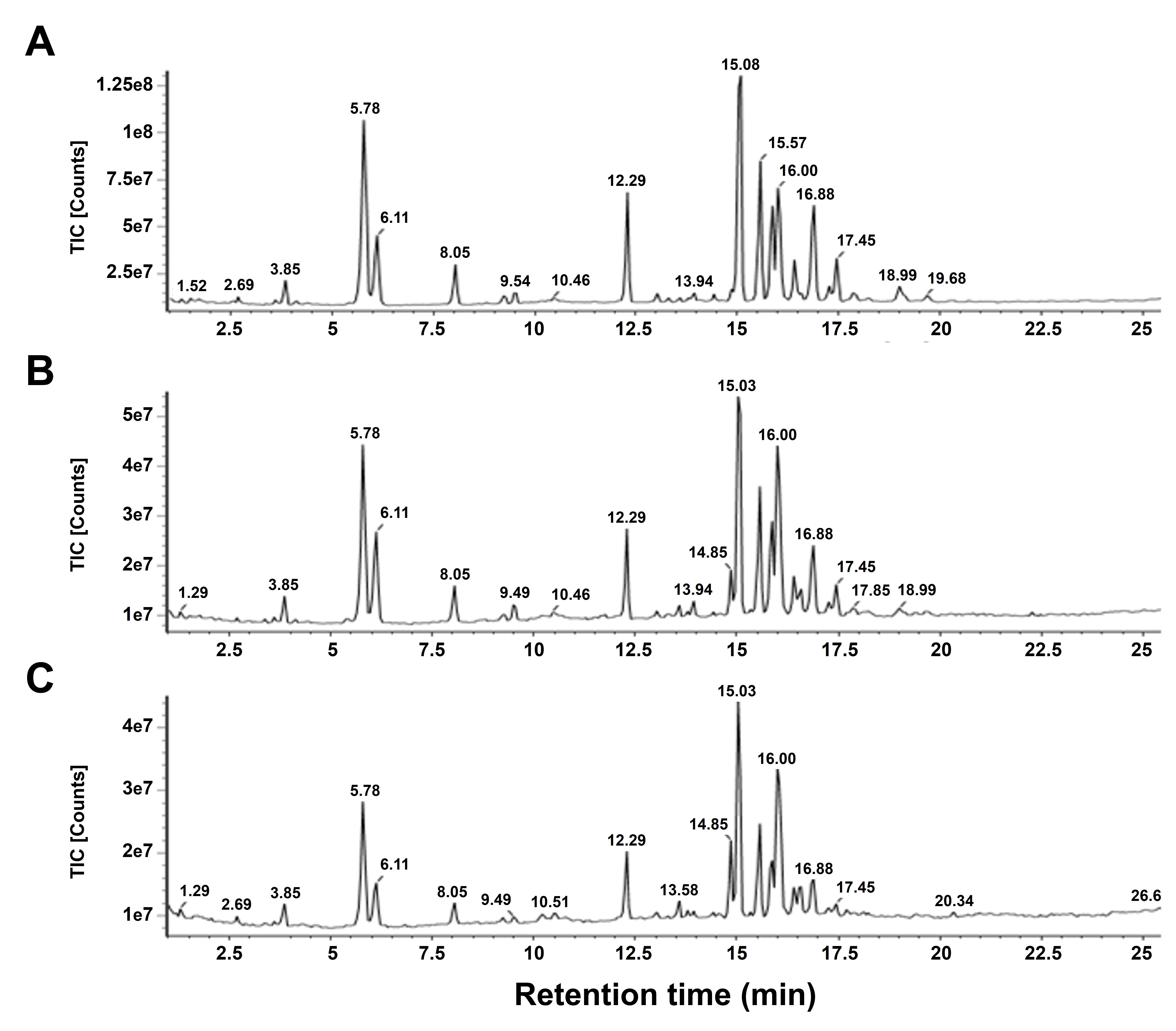

Supplement: Supplementary file 1 [file molecules-24-02381-s001.zip › FigureS2.jpg]

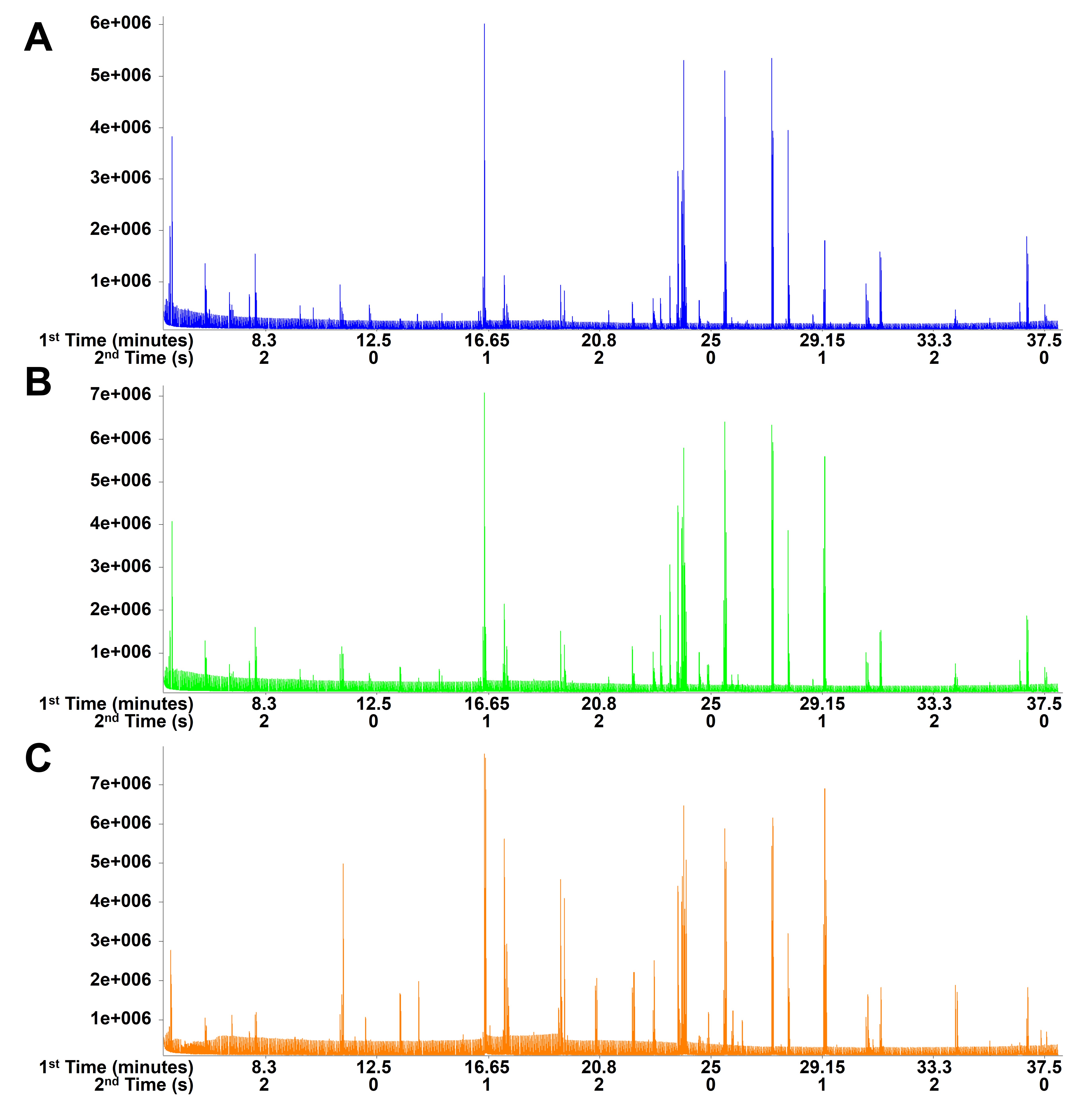

Supplement: Supplementary file 1 [file molecules-24-02381-s001.zip › FigureS3.jpg]
